# Can we achieve biomimetic electrospun scaffolds with gelatin alone?

**DOI:** 10.3389/fbioe.2023.1160760

**Published:** 2023-07-12

**Authors:** Elisa Roldán, Neil D. Reeves, Glen Cooper, Kirstie Andrews

**Affiliations:** ^1^ Department of Engineering, Faculty of Science and Engineering, Manchester Metropolitan University, Manchester, United Kingdom; ^2^ Research Centre for Musculoskeletal Science and Sports Medicine, Department of Life Sciences, Faculty of Science and Engineering, Manchester Metropolitan University, Manchester, United Kingdom; ^3^ School of Engineering, University of Manchester, Manchester, United Kingdom

**Keywords:** gelatin, electrospinning, nanofibers, solvent concentration, crosslinking, tissue engineering implants, MANOVA

## Abstract

**Introduction:** Gelatin is a natural polymer commonly used in biomedical applications in combination with other materials due to its high biocompatibility, biodegradability, and similarity to collagen, principal protein of the extracellular matrix (ECM). The aim of this study was to evaluate the suitability of gelatin as the sole material to manufacture tissue engineering scaffolds by electrospinning.

**Methods:** Gelatin was electrospun in nine different concentrations onto a rotating collector and the resulting scaffold’s mechanical properties, morphology and topography were assessed using mechanical testing, scanning electron microscopy and white light interferometry, respectively. After characterizing the scaffolds, the effects of the concentration of the solvents and crosslinking agent were statistically evaluated with multivariate analysis of variance and linear regressions.

**Results:** Fiber diameter and inter-fiber separation increased significantly when the concentration of the solvents, acetic acid (HAc) and dimethyl sulfoxide (DMSO), increased. The roughness of the scaffolds decreased as the concentration of dimethyl sulfoxide increased. The mechanical properties were significantly affected by the DMSO concentration. Immersed crosslinked scaffolds did not degrade until day 28. The manufactured gelatin-based electrospun scaffolds presented comparable mechanical properties to many human tissues such as trabecular bone, gingiva, nasal periosteum, oesophagus and liver tissue.

**Discussion:** This study revealed for the first time that biomimetic electrospun scaffolds with gelatin alone can be produced for a significant number of human tissues by appropriately setting up the levels of factors and their interactions. These findings also extend statistical relationships to a form that would be an excellent starting point for future research that could optimize factors and interactions using both traditional statistics and machine learning techniques to further develop specific human tissue.

## 1 Introduction

Gelatin is a natural polymer derived from the hydrolysis of collagen, the most abundant protein of the ECM. It is commonly used in biomedical, pharmaceutical and food packaging applications (Farris et al., 2010). Its low cost, high biocompatibility, hydrophilicity, biodegradability and bio-affinity make it attractive for the development of tissue engineered implants (Rashid et al., 2023). Moreover, gelatin contains lineal RGD (R arginine, G glycine, and D aspartate) integrin binding motif that promote cell adhesion and proliferation (Davidenko et al., 2016). Recently photo-curable gelatin-methacryloyl (GelMA) hydrogels are gaining more popularity in the research area to create 3D printed scaffolds for soft tissue engineered applications, due to their excellent biocompatibility, degradability and low cost (Pepelanova et al., 2018; Zhuang et al., 2019; Piao et al., 2021) However, despite the tremendous advance in the 3D printing scale ability, it is still in a developing process, with other manufacturing techniques such as the electrospinning currently being more appropriate for mimicking morphology of the extracellular matrix at the nanometer scale (Muldoon et al., 2022).

Scaffolds obtained from electrospinning of gelatin solubilized in various solvents have been characterized mechanically (Zha et al., 2012; Kalidas and Sumathi, 2023), morphologically (Huang et al., 2004; Choktaweesap et al., 2007; Song et al., 2008; Zha et al., 2012; Erencia et al., 2014; Erencia et al., 2015; Erencia et al., 2016; Maleknia and Majdi, 2014), chemically (Erencia et al., 2015; Erencia et al., 2016) and biologically through cytotoxicity texts (Zha et al., 2012; Erencia et al., 2015; Erencia et al., 2016; Kalidas and Sumathi, 2023). However, the results of all of these studies were not associated to a specific application, therefore they could not conclude whether electrospun scaffolds based on gelatin alone were suitable for manufacturing soft/hard tissue engineered implants.

Currently, electrospun gelatin scaffolds in combination with other polymers have been developed for wound healing applications (Ajmal et al., 2023; Lashkari et al., 2023; Zhang et al., 2023), nervous system tissue (Liu et al., 2021; Talebi et al., 2021; Zamanifard et al., 2023), dental applications (Sharifi et al., 2022; Acuña et al., 2023), bone tissue (Sun et al., 2022; Singh et al., 2023) and skin tissue (Baghersad et al., 2022; Farshi et al., 2022; Ghomi et al., 2023; Khalilimofrad et al., 2023), tendon implants (Wang et al., 2022; Xue et al., 2022) and vascular grafts (Zhu et al., 2020; Jiang et al., 2021; Fahad et al., 2023). All these studies have the characteristic of blending different synthetic polymers with gelatin to overcome shortcomings of both materials (Chong et al., 2007). The principal use of gelatin in these investigations was to improve cell adhesion, affinity and proliferation due to the content of RGD integrin binding sites (Gupta et al., 2009b; 2009a; Dhandayuthapani et al., 2010; Francis et al., 2010) while synthetic polymers were used to enhance the mechanical properties (Chong et al., 2007; Ghasemi-Mobarakeh et al., 2008; Kim et al., 2008; Gupta et al., 2009b; Wang et al., 2009; Yang et al., 2016). Moreover, some studies affirmed that electrospun scaffolds manufactured with single materials exhibited high fiber density that could reduce cell ingrowth (Li et al., 2005;; Kim et al., 2009). Gelatin is always used in combination with other polymers but there are possible advantages of using gelatin as a sole material which include simplifying the manufacturing process; to reduce the production costs due to the low cost of the gelatin and its solvents in comparison to others such as collagen; to minimize the hazard of using harmful organic solvents; and promote cell adhesion and proliferation. Despite its advantages, this polymer has four main disadvantages that should be addressed during its manufacturing process. The first disadvantage is the difficulty of working with electrospun aqueous gelatin solutions at room temperature (Huang et al., 2004; Erencia et al., 2015). These gelatin solutions become gel at temperatures below 30°C, which restricts the flow of solution through the needle and consequently the Taylor cone and fiber formation (Huang et al., 2004; Erencia et al., 2015). The second disadvantage is its high degradation rate that complicates the biological characterization of the scaffold and its use as an implant (Dalev et al., 2001). The third disadvantage is its poor mechanical properties with lower tensile stress and strain at rupture than some biological tissue such as cortical bone, tendon or ligament (Noyes et al., 1974; Martin et al., 2015; Mirzaali et al., 2016). The last disadvantage is the wide molecular weight distribution of gelatin. As mentioned previously, gelatin is a natural polymer derived from collagen, and its molecular weight can vary depending on the source and method of extraction (Ji et al., 2022). This variation in molecular weight could affect the physical and mechanical properties of the gelatin and may also affect its ability to form nanofibers via electrospinning.

To avoid the gelation process during electrospinning, alternative approaches with different solvents have been proposed in the literature. Fluorinated alcohols such as 2,2,2-triefluoroethanol (TFE) (Huang et al., 2004; Kim et al., 2013) or 1,1,1,3,3,3-hexafluoro-2-propanol (HIPF) (Horner et al., 2016); dilutions of phosphate buffer saline (PBS) in ethanol (Zha et al., 2012; Erencia et al., 2016); carboxylic acids such as formic acid or acetic acid (HAc) (Song et al., 2008; Erencia et al., 2014; Erencia et al., 2016; Maleknia and Majdi, 2014; Okutan et al., 2014; Siimon et al., 2015; Steyaert et al., 2016); mixtures of different solvents such as HAc and TFE (Choktaweesap et al., 2007), HAc and dimethyl sulfoxide (DMSO) (Choktaweesap et al., 2007), HAc and ethylene glycol (Choktaweesap et al., 2007), HAc and formamide (Choktaweesap et al., 2007) or HAc and ethyl acetate (Song et al., 2008) are the most common solvents used with gelatin. However, the use of organic solvents could affect the protein structure of the polymer and the cytotoxicity due to residual solvent in the scaffold (Yu et al., 2016). Additionally, the morphology, topography and mechanical properties of the scaffolds and their relationship with the cell viability, response and proliferation may be affected. Therefore, it is necessary to investigate how different concentrations of solvents affect these factors in gelatin-based electrospun scaffolds (Roldán et al., 2016b).

To address the limitations of having a high degradation rate and poor mechanical properties, the electrospun gelatin scaffolds must be crosslinked to create bonds between the protein chains and provide more stability and stiffness to the material. Many chemical crosslinking agents have been investigated in the literature, including glutaraldehyde, formaldehyde, glyceraldehyde, genipin, oxygen species, carbodiimide, diepoxy compounds, diisocyanates and dextran aldehydes (Kale and Bajaj, 2010). However, these crosslinkers reduce the number of free cell binding sites and with it the capacity of cell adhesion (Grover et al., 2012). The effect of different crosslinking techniques (immersion and vapor deposition) and concentrations of crosslinking agent (glutaraldehyde) on the degradability, morphology and topography of the scaffold needs to be further investigated.

The novelties of this work lie in understanding how changes in the solvent concentration affect the mechanical, morphological and topographical properties of the gelatin scaffolds and their ability to bio-mimic human tissue. In addition, how the use of a crosslinking agent changes the morphology of the nanofibers, and their network was evaluated to check the suitability of the polymer and crosslinker to manufacture different types of implants. To determine if gelatin-based electrospun scaffolds are suitable for tissue engineering applications, we explore the literature to determine design requirements for soft (Singh and Chanda, 2021) and hard tissue.

The aim of this work was to assess the suitability of gelatin as the sole material to manufacture tissue engineering scaffolds through the electrospinning technique. To outwork this aim, the morphology, topography and mechanical behavior of electrospun gelatin with different solvent and crosslinker concentrations was analyzed and compared with the design requirements for mechanical and morphological properties for soft and hard human tissues.

## 2 Materials and methods

### 2.1 Polymer solution

Gelatin powder type B from bovine skin (Bloom ∼225 g) was purchased from Sigma Aldrich (United Kingdom). Glacial acetic acid (Sigma Aldrich, United Kingdom), DMSO (Sigma Aldrich, United Kingdom) and distilled water (dH_2_O) were used as solvents.

Nine solutions were prepared with 25% w/v of gelatin dissolved in concentrations of HAc and dH_2_O of 3:1, 1:1 and 1:3, adding 0%, 5% and 10% of DMSO (Table 1). The electrospun gelatin scaffolds created with these nine solutions were mechanical and structural characterized to analyze the effect of solvent concentration.

**TABLE 1:** Performed studies to each gelatin electrospun scaffold.

Non-crosslinked scaffolds										Crosslinked scaffolds									
Morphology and topography			Mechanical properties				Statistical analysis			Morphology and topography			Degradation				Statistical analysis		
% gelatin	Hac/h?o	% DMSO	% gelatin	Hac/H2O	% DMSO	% gelatin		Hac/H2O	% DMSO	% gelatin	Hac/H2O	% DMSO		% gelatin	Hac/H2O	% DMSO	% gelatin	Hac/H2O	% DMSO
25	3:1	0	25	3:1	0	25		3:1	0	25				25			25		
		5			5				5										
		10			10				10										
	1:1	0		1:1	0			1:1	0		1:1	0			1:1	0		1:1	0
		5			5				5			5				5			5
		10			10				10			10				10			10
	1:3	0		1:3	0			1:3	0										
		5			5				5										
		10			10				10										

The crosslinker effect on the morphology, topography and degradation of the nanofibers was studied only in scaffolds created with 25% w/v of gelatin dissolved in 1:1 HAc/dH_2_O and 5% DMSO (Table 1), which was the solution that provided scaffolds with mechanical properties comparable to biological tissue such as trabecular bone (Figure 3).

### 2.2 Scaffold production

All the scaffolds used in both studies were fabricated with an electrospinning device (TL-01, NaBond, China) under the same set up of parameters, which allowed producing a stable Taylor cone and optimizing the quality of the scaffold manufacturing fibers free of defects. A 10 mL syringe was loaded with the solution to pump it with a flow rate of 2 mL/h through a 15 G needle. An electrostatic field was created applying 26 kV between the tip of the needle and the collector. The fibers were projected from the tip of the needle over a sheet of aluminum foil attached to a 15 cm diameter-rotating collector working at 1,300 rpm. The distance between the needle and the collector was set up to 11 cm. Each scaffold was manufactured at room temperature (25°C) and for 3 h spins time.

### 2.3 Chemical crosslinking

Only scaffolds created with 25% w/v of gelatin dissolved in 1:1 HAc/dH_2_O and 5% DMSO were crosslinked. Two crosslinking techniques were evaluated: immersion and vapor deposition.

For crosslinking the samples by immersion, three (0.5 × 0.5 cm) samples were individually placed in a 6-well plate covered by 2 mL of 25% GTA for 2 h.

For the crosslinking by vapor deposition, samples of 0.5 × 0.5 cm were cut, air-dried and place over a metallic mesh in a sealed desiccator to be crosslinked. The effect of the crosslinker over the electrospun fibers was tested with three different concentrations of GTA diluted in distillate water 2.5%, 5% and 25%. 25 mL of these solutions were poured separately in a Petri dish at the bottom of the desiccator and the samples were placed over a metallic mesh on top of the Petri dish to be exposed to glutaraldehyde vapor.

### 2.4 Degradation assay

Two samples of each concentration of GTA were air-dried for 24 h and the rest of the samples were placed in 6 well dishes with 4 mL of phosphate-buffered saline buffer (PBS) in each well and left in an incubator at 37°C and 4% CO_2_ in order to test their degradability with time in an environment representative of the human body. A total of 42 samples, two samples for each concentration, were left for 1, 5, 7, 14, 21 and 28 days in PBS and then dried air for 24 h in a fume hood.

### 2.5 Scaffold/fiber characterization

#### 2.5.1 Morphology of the fibers

A SC7640 sputter coater (Quorum Technologies Ltd., Kent, United Kingdom) was used to coat the crosslinked and non-crosslinked samples with gold prior to their visualization with a field emission scanning electron microscope Zeiss Supra 40 (FE-SEM, Carl Zeiss SMT Ltd., Cambridge, United Kingdom). The intensity used for coating the samples was 20 mA, the voltage 0.8 kV and the duration of the coating was 120 s, which provides a coating thickness of 32.6 nm, following equipment specifications. Scanning electron microscope (SEM) images were taken at approx. 6 mm working distance, 3 kV and with ×50,000 magnifications. Fiber diameter (Ø fiber) and inter-fiber separation (Int.sep.) were determined with AxioVision SE64 Rel. 4.9.1 (Carl Zeiss SMT Ltd., Cambridge, United Kingdom) by measuring 40 fibers per sample. The inter-fiber separation was defined as the maximum horizontal distance between two fibers that belong to the same pore. A pore was defined as the void space constituted by fibers (normally four fibers) that intersect one with each other and are located on the same layer of fibers. Fiber diameter and inter-fiber separations were measure in the same way in dry conditions and during the degradation assay.

#### 2.5.2 Topography of the scaffold

The average roughness of the crosslinked and non-crosslinked scaffolds was measured by taking six white light interferometry images per sample using an interferometer from ZeGage (Zygo Corporation, United Kingdom) following a previous study (Accardi et al., 2013). This equipment allowed high-precision 3D metrology of surface features to be obtained, determining the topography of scaffolds through measuring characteristics such as maximum peak-to-valley profile height or average roughness. The average roughness was compared between samples with different GTA concentrations and days in PBS in order to understand how the crosslinking and the incubation in PBS affected to the topography of the samples.

#### 2.5.3 Mechanical behavior of the scaffold

Four samples were mechanically analyzed for each non-crosslinked scaffold following a previous study (Huang et al., 2004). The samples were removed with a dog-bone cutting die (25 × 4 mm, test length x width), and their thickness measured three times with a digital and an analogue caliper in order to check the consistency of the measurements and corroborated with SEM images; samples were attached to a cardboard frame to aid in the alignment of the sample in the tensometer (Instron H10KS, United States) and a quasi-static uniaxial tensile test was performed until failure with a 1 mm/min test speed measured with a 100 N load cell (Balan et al., 2016; Salifu et al., 2017; Ghosal et al., 2018). It is worth noticing that after observing the cross-section area under a SEM, we noticed that mainly vertical packed fibers were created generating a low thickness scaffold; therefore, we assumed that the inter-fiber separations on the cross-sectional area were neglectable. Mechanical properties such as Young’s modulus, tensile strength and strain at break were determined for each sample and statistically analyzed in order to find a relationship between mechanical and morphological properties.

### 2.6 Statistical analysis

Mean, standard deviation and standard error of the mean (Std Error) of the 360 observations for each structural and mechanical properties were calculated.

In order to compare these properties between scaffolds made with different solvents concentrations and evaluate the degradation of the scaffold, a complete (type 1) multivariate analysis of variance (MANOVA) with a 95% confidence was performed. Independent variables were the concentration of HAc, dH_2_O and DMSO and the dependent variables were the fiber diameter, inter-fiber separation, Young’s modulus, ultimate tensile strength and strain at break. Coefficient of determination, Mean Squared Error (MSE) and Root Mean Square Error (RMSE) were calculated to assess the goodness of the fit of the model. ɳ^2^ was calculated to study the importance of each factor and their interaction.

Linear regressions were performed to calculate the relationship between morphological, topographical and mechanical properties of each scaffold and solvent concentrations. The degradability of the scaffolds was also statistically studied through linear regressions that related fiber diameter, inter-fiber separation and average roughness of the scaffolds with the number of days that the scaffold was immersed in PBS.

All statistical analyzes were conducted using SPSS (IBM Inc., United States).

Table 1 shows a summary of the scaffolds used in each study.

### 2.7 Extracellular matrix design requirements

A literature search was performed using the database Scopus and search terms such as “mechanical properties,” “Young’s modulus,” “ultimate tensile strength,” “strain at break” and different hard and soft tissue including “trabecular bone,” “cortical bone,” “skin,” “muscle,” “tendon,” “ligament,” “ACL,” “blood vessels,” “nervous tissue,” “nasal tissue” or “oral tissue” among other human tissues. Mechanical properties of tissues such as esophagus, stomach, liver, gallbladder or kidney were found in (Singh and Chanda, 2021). This literature review helped to determine the key mechanical properties of common tissues. The data from the literature was used to benchmark the results from the experimental part of this study and to qualitatively and quantitatively evaluate the possibility of using gelatin alone in electrospun scaffolds for extracellular matrix replacements.

## 3 Results and discussion

### 3.1 How different concentrations of solvents affect the morphology, topography and mechanical properties of the scaffolds

**FIGURE 1:**
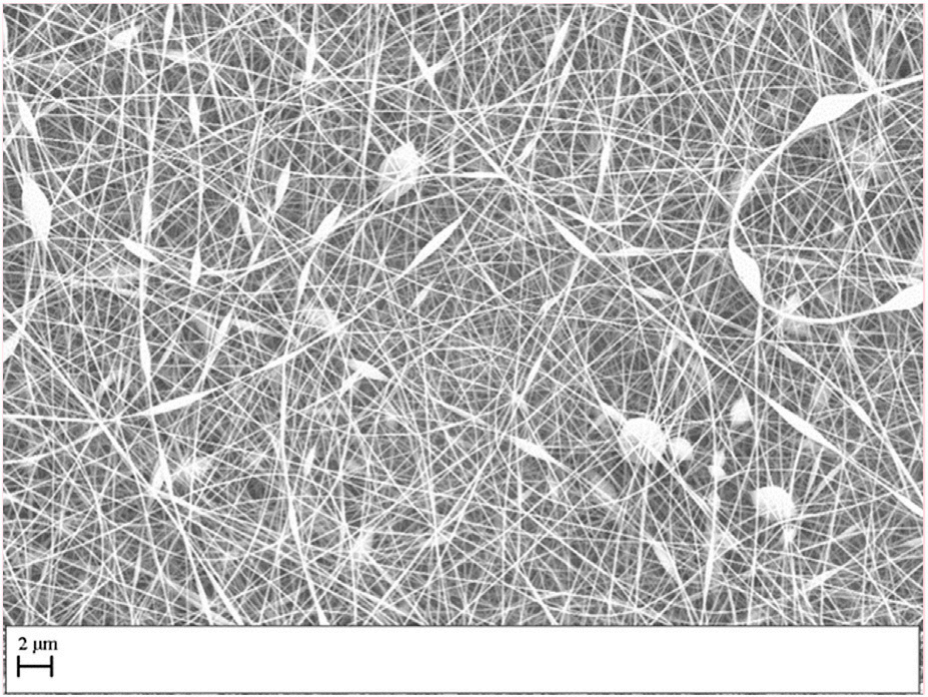
Electrospun scaffold performed with a concentration of gelatin of 20% w/v diluted in HAc and dH_2_O.

Initial experiments determined an optimum concentration of gelatin of 25% w/v diluted in HAc, dH_2_O and DMSO, concentrations above this value increased the viscosity of the solution making it difficult for electrospinning; below this concentration, defects on the fibers, bead formation and beaded fibers were observed, reducing the mechanical properties of the scaffold and likely impeding cell migration (Figure 1).

HAc and DMSO were selected in this study among the large variety of solvents used for gelatin. The principal reasons why HAc was selected were: because HAc enables optimization of the electrospinning process avoiding gelation; HAc was reported to pose fewer environmental and health risks than normal organic solvents used with gelatin (Avossa et al., 2022); and it was demonstrated that the cell viability was higher than 90% in gelatin-based electrospun scaffolds created with 25% HAc and 80% cell viability was found in gelatin-based electrospun scaffolds created with 75% HAc (Erencia et al., 2015). DMSO was selected due to it favors the creation of smoother fibers free of defects (Choktaweesap et al., 2007), what might increase the mechanical properties of the scaffold.

**FIGURE 2:**
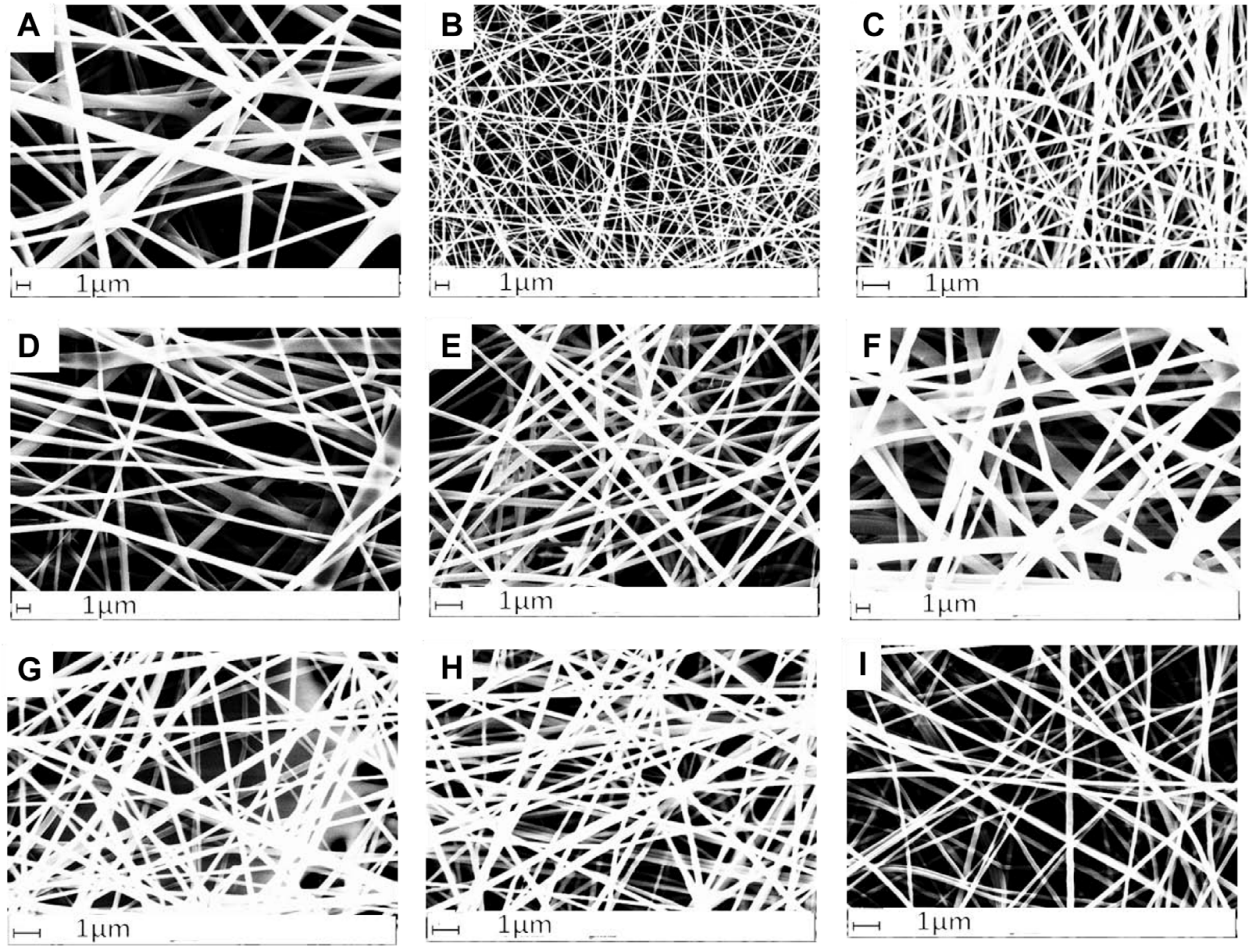
Electrospun scaffold obtained with a concentration of gelatin 25% w/v dissolved in concentrations **(A)** 3:1 HAc/dH_2_O and 0% DMSO, **(B)** 1:1 HAc/dH_2_O and 0% DMSO, **(C)** 1:3 HAc/dH_2_O and 0% DMSO, **(D)** 3:1 HAc/dH_2_O and 5% DMSO, **(E)** 1:1 HAc/dH_2_O and 5% DMSO, **(F)** 1:3 HAc/dH2O and 5% DMSO, **(G)** 3:1 HAc/dH_2_O and 10% DMSO, **(H)** 1:1 HAc/dH_2_O and 10% DMSO, **(I)** 1:3 HAc/dH_2_O and 10% DMSO.

Figure 2 shows the morphology of the electrospun scaffolds obtained with the nine solutions prepared with 25% w/v of gelatin dissolved in concentrations of HAc and dH_2_O of 3:1, 1:1 and 1:3, adding 0%, 5% and 10% of DMSO.

The MANOVA revealed that fiber diameter, inter-fiber separation and mechanical properties were significantly affected by the concentration of HAc, dH_2_O, DMSO and their interaction (*p* < 0.001), with significant differences found between scaffolds manufactured with different solvents concentrations and the scaffold parameters (Table 2).

**TABLE 2:** Level of significance of factors and their interactions.

Dependent variables	Significance of independent variables and their interactions				
	HAc	dH20	DMSO	HAc X DMSO	DMSO X dH_2_0
Diameter	0.000000	0.001473	0.000000	0.0000000	0.000000
Inter-fiber separation	0.000000	0.011155	0.0001166	0.002060	0.058484
Ultimate tensile strength	0.000000	0.003100	0.000000	0.000000	0.000302
Young’s modulus	0.000000	0.000000	0.000000	0.000000	0.000000
Strain at break	0.000000	0.000000	0.000000	0.000000	0.194235
*** Significant >0.001					

The goodness of fit of the model, Mean Squared Error (MSE) and Root Mean Square Error (RMSE) are presented in Table 3, below.

**TABLE 3:** Coefficient of determination, MSE and RMSE for each dependent variable.

	Dependent variables				
	Diameter	Inter-fiber separation	Ultimate tensile strength	Young’s modulus	Strain at break
R^2^	0.728	0.483	0.870	0.928	0.670
MSE	0.03	1.64	0.28	551.64	0.16
RMSE	0.182	1.281	0.532	23.487	0.394

The concentration of HAc provides more variability than the DMSO concentration, to the diameter of the fibers (partial ɳ^2^ = 0.677 and 0.158 respectively). It similarly occurs with the inter-fiber separation; therefore, the concentration of HAc is more relevant than the concentration of DMSO on the morphology of the scaffolds. However, the influence of DMSO is higher than the concentration of HAc on the prediction of the mechanical properties. The importance of the three factors on the general variability is shown in Table 4.

**TABLE 4:** MANOVA: Importance of each factor and interactions on the prediction of the dependent variables (ɳ^2^).

Dependent variables		Factors and interactions					Total	%
		HAc	dH_2_O	DMSO	HAc * DMSO	dH_2_O * DMSO		
Morphology	Diameter	0.677	0.028	0.158	0.164	0.149	1.18	67.55
	Inter-fiber separation	0.448	0.018	0.049	0.034	0.016	0.56	32.45
	Total	1.125	0.045	0.207	0.198	0.164	1.740	23.99
	%	64.67	2.61	11.92	11.36	9.44	100.00	
Mechanical properties	Ultimate tensile strength	0.496	0.024	0.824	0.488	0.044	1.88	34.04
	Young’s modulus	0.229	0.337	0.903	0.710	0.281	2.46	44.63
	Strain at break	0.066	0.298	0.539	0.264	0.009	1.18	21.34
	Total	0.791	0.659	2.266	1.462	0.334	5.512	76.01
	%	14.35	11.96	41.12	26.52	6.06	100.00	
Total		1.916	0.705	2.474	1.659	0.498	7.25	100.00
%		26.42	9.72	34.11	22.88	6.87	100.00	

**FIGURE 3:**
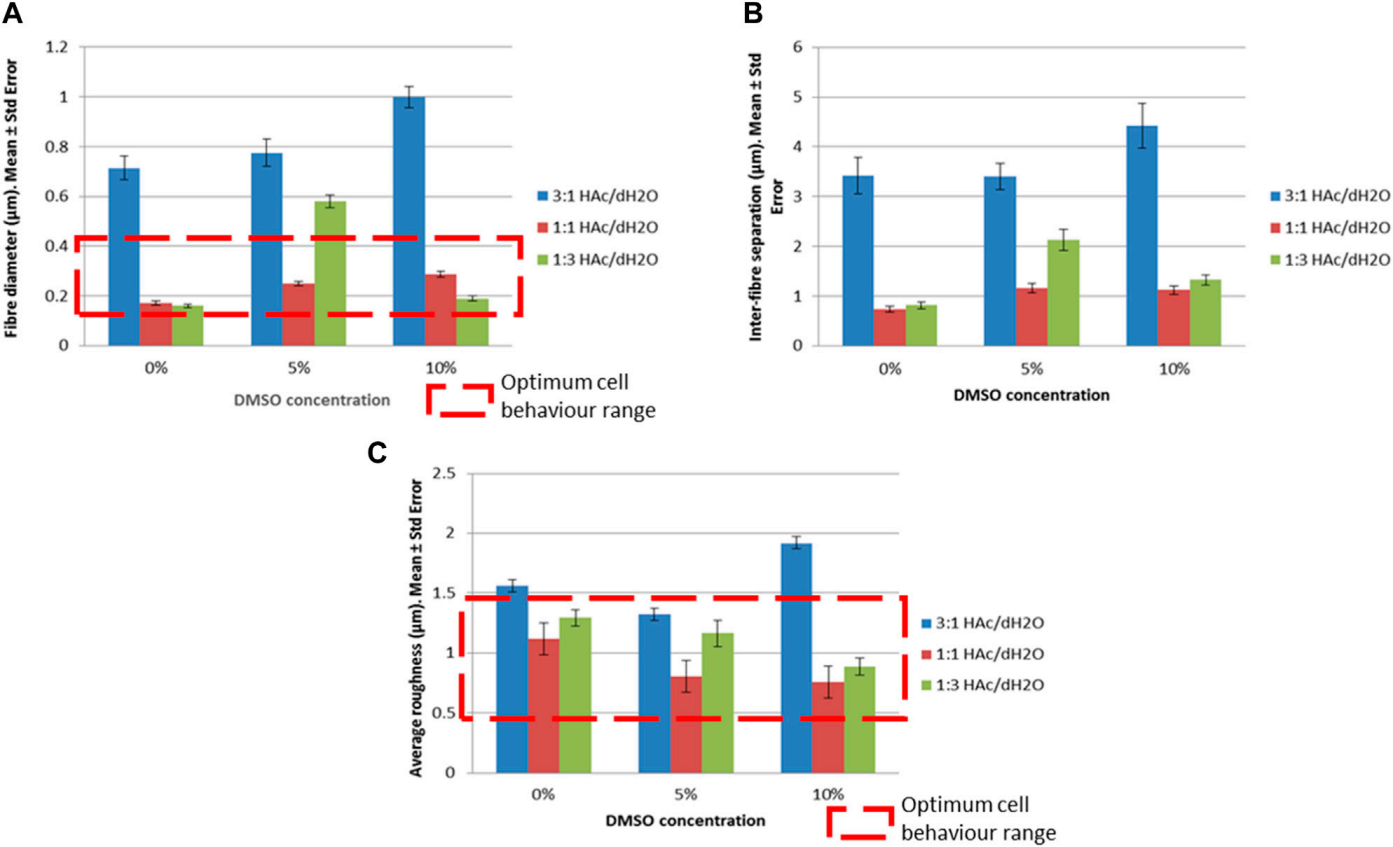
Morphological and topographical properties for different concentrations of HAc and DMSO and their optimum cell behavior range **(A)** Fiber diameter (μm), **(B)** Inter-fiber separation (μm), **(C)** Average roughness (μm).

The relevance of HAc against the concentration of DMSO on the morphology of the scaffolds can be observed in Figures 3A, B where the 3:1 HAc/dH_2_O concentration provided greater fiber diameter and inter-fiber separation than all DMO concentration. This was also corroborated by Erencia et al. (2015) in 2015 where solutions of gelatin with concentrations of 200, 250, 300, 350, and 400 mg/mL (kg/m3) were prepared using acetic acid at different concentrations [25, 50, and 75% (v/v)] as solvent. Erencia et al. (2015) proved that the viscosity of the polymer and consequently the thickness of the fibers increased when the HAc concentration was increased. Another outcome observed from an increase of the polymer viscosity was that the fibers were more aligned when the HAc and DMSO concentration was increased.

A stepwise regression method was used to optimize the number of regression variables. And the non-collinearity of the regression variables was proved. Multiple regression models revealed that fiber diameter and inter-fiber separation increased when the concentration of HAc and DMSO increased, with a calculated F highly significant (*p* < 0.001) for the regression variables.

The regression models obtained for predicting the diameter of the fibers 1) and inter-fiber separation 2) were:

$$Fiber\;diameter\;\left(µm\right)=-0.075+1.432\;x\;DMSO+0.276\;x\;HAc$$ (1)

$$Interfiber\;separation\;\left(µm\right)=-0.297+6.012\;x\;DMSO+1.21\;x\;HAc$$ (2)

A relationship between fiber diameter and inter-fiber separation was also observed; the greater the fiber diameter, the greater the inter-fiber separation was obtained. These findings agree with the thesis of Joseph Lenning Lowery (Massachusetts Institute of Technology, 2009) (Lowery, 2009) where it was proved that the packing of the fibers was more dense when the fibers were thinner. The scaffolds with the largest fiber diameter and inter-fiber separations were those manufactured with 3:1 HAc/dH_2_O and 10% DMSO, as it is observed in Figures 3A, B. However, no evidence of high fiber density was observed in these gelatin electrospun scaffolds, therefore no negative effect on the cell ingrowth is expected on electrospun scaffolds manufactured with a single material.

**FIGURE 4:**
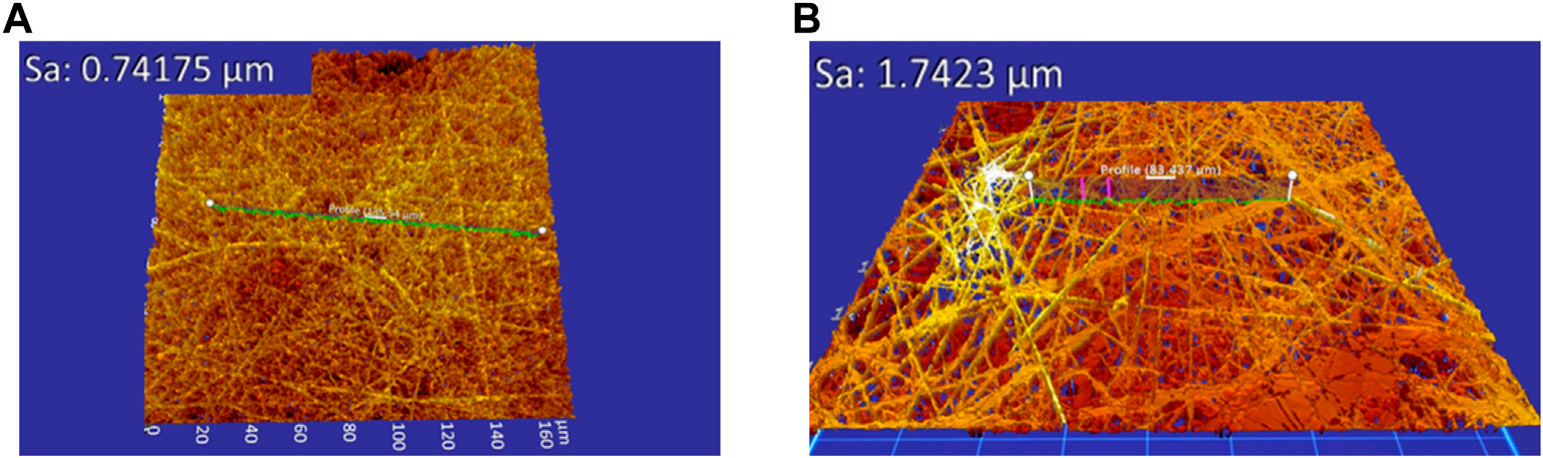
Average roughness of the electrospun scaffolds **(A)** 1:1 HAc/dH2O and 5% DMSO (μm), **(B)** 3:1 HAc/dH2O and 10% DMSO.

Analysing the roughness in relation to the acetic acid concentration, the minimum roughness was shown as 1:1 HAc/dH_2_O and the maximum as 3:1 HAc/dH_2_O (Figure 3C; Figure 4). The average roughness of the scaffold tended to decrease when the DMSO concentration was increased, allowing the creation of smoother fibers with an absence of beads, a fact that was also corroborated by Choktaweesap et al. (2007). Choktaweesap et al. (2007) studied the effect of a single solvent system (glacial acetic acid) and mixed solvent systems (glacial acetic acid in combination with different solvents: 2,2,2-trifluoroethanol (TFE), dimethyl sulfoxide (DMSO), ethylene glycol (EG) and formamide F)) on the morphology and fiber diameter. Choktaweesap et al. (2007) concluded that the addition of TFE, DMSO, EG, or F as co-solvent helped to improve the electrospinnability of the resulting gelatin solution. Moreover, DMSO and, EG contributed to the formation of smooth gelatin fibers with reduced diameters in comparison to fibers created with acetic acid as the sole solvent.

In this case, the regression model that predicts the average roughness 3) related to the solvent concentration was:

$$Average\;roughness\;\left(µm\right)=1.756-0.014\;x\;DMSO-0.242\;x\;HAc$$ (3)

A 90% cell viability was reported with gelatin-based electrospun scaffolds with diameter of fibers between 97 ± 12 nm and 429 ± 68 nm (Erencia et al., 2016), values comparable to the reported in the present study (Figure 3A).

Andrews and Hunt (2008) reported that cellular behavior is influenced by the scaffold topography. Values of roughness between 0.3 µm and 1.2 µm exhibited high percentage of cell coverage and cell spreading after 28 days of being seeded (Andrews and Hunt, 2008). Most of the scaffolds manufactured in the current study presented values of roughness in the same range as it was reported by Andrews & Hunt (2008). Therefore, these results (Figure 3C) suggest that a high percentage of cell coverage and cell spreading is expected with the scaffolds produced in this study.

Figure 4 shows the average roughness of the electrospun scaffolds obtained with 1:1 HAc/dH2O and 5% DMSO, and 3:1 HAc/dH2O and 10% DMSO.

Generalized and mixed statistical models and machine learning methods, such as decision trees and artificial neural network, could be utilized in following studies to allow classification and optimize the models, as demonstrated in a recent research study (Roldán et al., 2023).

Mechanical properties were significantly affected by the concentration of DMSO (*p* < 0.001). Scaffolds with 1:1 HAc/dH_2_O exhibited the greatest tensile strength and strain at break at each DMSO concentration (Table 5), due to the fact that the inter-fiber separations of those scaffolds were the lowest allowing the creation of denser packing of fibers.

**TABLE 5:** Mechanical properties of the developed gelatin-based electrospun scaffolds.

Concentration of the solvents	Young’s modulus (MPa)			Ultimate tensile strength (MPa)			Strain at break (%)		
	Mean ± SD	Max	Min	Mean ± SD	Max	Min	Mean ± SD	Max	Min
3:1 HAc/dH_2_O and 0% DMSO	122.395 ± 12.110	141.601	99.631	1.859 ± 0.345	2.221	1.121	2.042 ± 0.678	3.160	0.924
1:1 HAc/dH_2_O and 0% DMSO	221.848 ± 22.625	261.924	185.298	4.524 ± 0.601	5.462	3.373	2.447 ± 0.479	3.140	1.855
1:3 HAc/dH_2_O and 0% DMSO	275.098 ± 22.570	308.968	236.587	4.388 ± 0.299	4.698	3.852	1.905 ± 0.076	2.143	1.854
3:1 HAc/dH2O and 5% DMSO	359.287 ± 30.373	408.870	315.048	3.882 ± 0.918	4.962	2.156	1.078 ± 0.116	1.573	1.060
1:1 HAc/dH_2_O and 5% DMSO	300.961 ± 18.859	339.675	272.524	4.639 ± 0.704	5.678	3.062	2.243 ± 0.196	2.943	2.047
1:3 HAc/dH_2_O and 5% DMSO	292.654 ± 7.851	306.273	278.997	4.620 ± 0.665	5.553	3.563	1.710 ± 0.559	2.442	1.103
3:1 HAc/dH_2_O and 10% DMSO	136.567 ± 30.284	180.945	83.871	1.617 ± 0.267	1.920	1.014	1.332 ± 0.124	1.777	1.246
1:1 HAc/dH_2_O and 10% DMSO	110.529 ± 37.298	184.466	51.957	1.933 ± 0.285	2.303	1.384	1.790 ± 0.416	1.847	0.831
1:3 HAc/dH_2_O and 10% DMSO	189.225 ± 12.194	204.994	166.699	1.315 ± 0.213	1.912	0.960	0.654 ± 0.384	1.261	0.060

The tensile strength can be predicted with the following regression model 4):

$$Tensile\;strength\;\left(MPa\right)=2.456-0.166\;x\;DMSO+0.788\;x\;HAc$$ (4)

**FIGURE 5:**
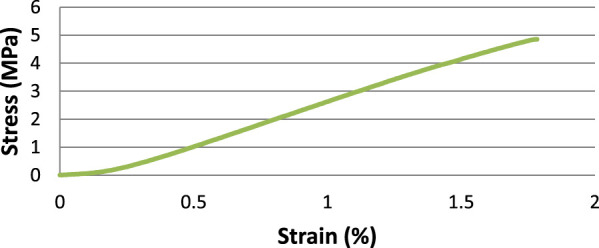
Stress-Strain curve for a 1:1 HAc/dH_2_O and 5% DMSO scaffold.

Stress-strain curves were plotted for each tested sample. The J-shape characteristic of gelatin-based electrospun scaffolds (Huang et al., 2004) and biological soft tissue (Cruz et al., 2014), was found in each gelatin-based electrospun scaffolds manufactured. This J-shape is typically shown to have a toe-region where the fibers start to straighten in the direction of the applied load and recover their structure once load has been removed, a linear region where the Young’s modulus was calculated for each sample, and the yield region. A typical stress-strain curve shown in Figure 5.

A literature review of the mechanical properties of different biological tissue was performed to evaluate the suitability of gelatin-based electrospun scaffolds for different tissue engineering applications. Mechanical properties of hard and soft human tissue can be found in Table 6.

**TABLE 6:** Mechanical properties of the human tissues with similarities in bold to the developed gelatin-based electrospun scaffolds.

Tissue name	Sample location/testing method	Young’s modulus (MPa)			Ultimate tensile strength (MPa)			Strain at break (%)			References
		Mean ± SD	Max	Min	Mean ± SD	Max	Min	Mean ± SD	Max	Min	
Hard tissue											
Cortical bone tissue		18,200 ± 1900	20,100	16,300	92.9 ± 10.1	103	82.8				Mirzaali et al. (2016)
Trabecular bone tissue		**301 ± 100**	401	201	**2.2 ± 0.7**	2.9	1.5	**1.6 ± 0.3**	1.9	1.3	Kopperdahl and Keaveny (1998)
		**489 ± 331**	820	158	**2.22 ± 1.42**	3.64	0.8	**1.59 ± 0.33**	1.92	1.26	Røhl et al. (1991)
		**272 ± 195**	467	77	**2.54 ± 0.62**	3.16	1.92				Goldstein (1987)
		**421 ± 208**	629	213	**5.6 ± 3.8**	9.4	1.8				Kuhn et al. (1989)
Soft Tissue											
Skin tissue		**83.3 ± 34.9**	118.2	48.4	21.6 ± 8.4	30	13.2				Ní Annaidh et al. (2012)
	Parallel to the fibers	**160.8 ± 53.2**	214	107.6	28.0 ± 5.7	33.7	22.3				Ottenio et al. (2015)
	Perpendicular to the fibers	**70.6 ± 59.5**	130.1	11.1	15.6 ± 5.2	20.8	10.4				Ottenio et al. (2015)
	Abdominal	18.8	18.8	18.8	**1–24**	24	1				Jansen and Rottier, (1958); Dunn and Silver, (1983), Joodaki and Panzer, (2018)
	Middle back parallel to the fibers	**112.5**	112.5	112.5	17–28	28	17				Ní Annaidh et al. (2012) Joodaki and Panzer, (2018)
Muscle tissue	Temporal muscle	1.58 ± 0.64	2.22	0.94	0.26 ± 0.11	0.37	0.15				Zwirner et al. (2020)
Connective tissues	Human tendons	1,000–1,500	1.5	1	100–140	100	140				(Martin et al., 2015) (Biewener, 2008)
	Human tendons	504–660	660	504	157.4 ± 33.8	191.2	123.6	18.8 ± 4.1	18.8	18.8	Johnson et al. (1994); Louis-Ugbo et al. (2004) Thorpe et al. (2012)
	Achilles’ tendon	870 ± 200	1,070	670							Lichtwark and Wilson (2005)
	Achilles’ tendon	1,160 ± 150	1,310	1,010							Maganaris and Paul (2002)
	Anterior cruciate ligament	**111**	111	111	38	38	38	44	44	44	Noyes and Grood (1976)
	Patellar ligament	**225**	225	225							Butler et al. (1986)
	Ankle ligaments	**260**	260	260							Siegler et al. (1988)
Cardiovascular tissue	Blood vessels	2–6	2	6				50	50	50	Ebrahimi (2009)
	Blood vessels				**4.3–6.3**	6.3	4.3	59–120	59	120	Camasão and Mantovani (2021)
	Pulmonary valve	16.05 ± 2.02	18.07	14.03	**2.78 ± 1.05**	3.83	1.73				Stradins et al. (2004)
	Aortic valve	15.34 ± 3.84	19.18	11.5	**1.74 ± 0.29**	2.03	1.45				Stradins et al. (2004)
Nervous tissue		0.6	0.6	0.6				40	40	40	Borschel et al. (2003) Topp and Boyd, (2006)
Nasal tissue	Nasal periosteum				**3.88**	3.88	3.88				Zeng et al. (2003)
Oral tissues	Gingiva	**37.36 ± 17.4**	**54.76**	19.96	**3.81 ± 0.9**	4.71	2.91				Choi et al. (2020)
	Hard palate	18.13 ± 4.51	22.64	13.62	**1.70 ± 0.9**	2.6	0.8				Choi et al. (2020)
	Buccal mucosa	8.33 ± 5.78	14.11	2.55	**1.54 ± 0.5**	2.04	1.04				Choi et al. (2020)
Esophagus					**1.2**	1.2	1.2				Egorov et al. (2002)
Stomach	Axial loading				0.7	0.7	0.7				Egorov et al. (2002)
Liver tissue					**1.85 ± 1.18**	3.03	0.67				Brunon et al. (2010)
Gallbladder	Axial loading	0.64 ± 0.02	0.66	0.613	**1.24 ± 0.99**	2.23	0.24				Karimi et al. (2015)
Kidney tissue	Capsular membrane	**41.5**	41.5	41.5	9.0 ± 2.9	11.9	6.1				Snedeker et al. (2005)
Small intestinal tissue	Fresh intestinal samples	2.69 ± 0.37	3.06	2.32	0.9	0.9	0.9				Bourgouin et al. (2012)
Colon	Dynamic loading	3.16 ± 1.89	5.05	1.27							Massalou et al. (2019)
Urinary bladder		1.9 ± 0.2	2.1	1.7	0.9 ± 0.1	1	0.8				Martins et al. (2011a)
Uterus tissue	Round ligament	9.1–14.0	14	9	**4.1**	4.1	4.1				Baah-Dwomoh et al. (2016)
	Uterosacral ligament	0.75–29.8	0.75	30	**4**	4	4				Baah-Dwomoh et al. (2016)
Vaginal tissue	Longitudinal	6.2 ± 1.5	7.7	4.7	**2.3 ± 0.5**	2.8	1.8				Martins et al. (2011b)
	Transversal	5.4 ± 1.1	6.5	4.3	**2.6 ± 0.9**	3.5	1.7				Martins et al. (2011b)

Cardiovascular and nervous tissue exhibited higher strain at break (50% (Ebrahimi, 2009) and 40% (Borschel et al., 2003; Topp and Boyd, 2006) respectively) and lower Young’s modulus (2–6 MPa (Ebrahimi, 2009) and 0.6 MPa (Borschel et al., 2003; Topp and Boyd, 2006) respectively) than the manufactured scaffolds.

Human abdomen skin exhibited values of ultimate tensile strength on the same range as the scaffolds (1–24 MPa (Jansen and Rottier, 1958; Dunn and Silver, 1983; Joodaki and Panzer, 2018)); however, the Young’s modulus of the skin in this part of the body is lower than the one observed on the scaffolds (18.8 MPa (Dunn and Silver, 1983; Joodaki and Panzer, 2018)). Only the Young’s modulus increases until values of 112.5 MPa (Ní Annaidh et al., 2012; Joodaki and Panzer, 2018) in the middle back, being comparable to the values observed on the manufactured scaffolds with 1:1 HAc/dH_2_O and 10% DMSO (maximum average tensile strength of 1.9 ± 0.6 MPa, Young’s Modulus of 110.5 ± 62.1 MPa and strain at break of 1.8% ± 0.3%).

The values of Young’s Modulus (1.58 ± 0.64 MPa) and ultimate tensile strength (0.26 ± 0.11 MPa) of muscle tissue were lower than the developed scaffolds (Zwirner et al., 2020).

Human tendons exhibited higher ultimate tensile strength, Young’s Modulus and strain at break than the scaffolds created (Young’s Modulus of 504–660 MPa (Johnson et al., 1994; Louis-Ugbo et al., 2004), strain at break of 18.8% ± 4.1% (Thorpe et al., 2012) and maximum tensile strength of 157.4 ± 33.8 MPa (Thorpe et al., 2012)).

The natural anterior cruciate ligament (ACL) has a Young’s modulus of approx. 111 MPa (Noyes and Grood, 1976), 38 MPa of maximum tensile strength and 44% of strain at break. *In-vivo* studies revealed that the maximum tensile strength exhibited by the ACL in a daily activity such as walking is approximately 17.6 MPa (Roldán et al., 2017) and the maximum strain for activities like walking or climbing stairs is approx. 13% and 8.6% respectively (Roldán et al., 2016a; 2017). Therefore, the material properties of the gelatin-based electrospun scaffolds did not reach the specifications needed for daily activities.

Zeng et al. (2003) reported values of ultimate tensile strength of nasal periosteum of 3.88 MPa (Zeng et al., 2003), comparable to 3.882 ± 0.918 MPa obtained with electrospun gelatin-based scaffolds created with 3:1 HAc/dH_2_O and 5% DMSO.

Choi et al. (2020) studied the mechanical properties of different tissues belonging to the oral cavity. Gingiva, hard palate and the buccal mucosa exhibited values of ultimate tensile strength comparable to the manufactured scaffolds (3.81 ± 0.9 MPa, 1.70 ± 0.9 MPa and 1.54 ± 0.5 MPa respectively (Choi et al., 2020)). However, only the gingiva reached a Young’s modulus similar to the one obtained with the scaffolds created with 1:1 HAc/dH_2_O and 10% DMSO.

The ultimate tensile strength of the esophagus was 1.2 MPa (Egorov et al., 2002) and Karimi et al. (2015) reported values of ultimate tensile strength for the gallbladder of 1.24 ± 0.99 MPa (Karimi et al., 2015), both values were comparable to the ultimate tensile strength obtained with gelatin solutions of 1:3 HAc/dH_2_O and 10% DMSO.

Brunon et al. (2010) found values of ultimate tensile strength for liver tissue of 1.85 ± 1.18 MPa (Brunon et al., 2010), similar to the values obtained with our gelatin-based scaffolds developed with 3:1 HAc/dH_2_O and 0% DMSO.

Round and uterosacral ligaments presented values of ultimate tensile strength of 4.1 MPa and 4 MPa respectively (Baah-Dwomoh et al., 2016), similar to the ones got with gelatin solutions of 1:3 HAc/dH_2_O and 0% DMSO. However, the Young’s Modulus of the human tissue were significantly different to the developed scaffolds. A similar issue occurs with the vaginal tissue, ultimate tensile strength of this tissue [2.3 ± 0.5 MPa in the longitudinal axes and 2.6 ± 0.9 MPa in the transversal axes (Martins et al., 2011b)] is comparable with gelatin solutions with concentrations of 3:1 HAc/dH_2_O and 5% DMSO; however, the Young’s Modulus of the vaginal tissue is significantly lower than the one obtained with the scaffolds.

Cortical bone tissue presented values of Young’s Modulus of 18.2 ± 1.9 GPa (Mirzaali et al., 2016) and maximum tensile strength of 92.9 ± 10.1 MPa (Mirzaali et al., 2016); however, these properties for human trabecular bone tissue are 301 ± 100 MPa and 2.2 ± 0.7 MPa respectively, and the strain at break was 1.6% ± 0.3% (Kopperdahl and Keaveny, 1998). Therefore, electrospun gelatin scaffolds with 1:1 HAc/dH_2_O and 5% DMSO resulted the most comparable for use as tissue engineering trabecular bone, due to their mechanical properties were: maximum average tensile strength of 4.6 ± 0.9 MPa, Young’s Modulus of 300.9 ± 32.6 MPa and strain at break of 2.2% ± 0.3%. Further research has to be done to determine the mechanical behavior of these scaffolds under shear and compression forces and after being crosslinked. Moreover, the feasibility of manufacturing 3D trabecular bone implants through gelatin-based electrospun scaffolds has to be evaluated.

**FIGURE 6:**
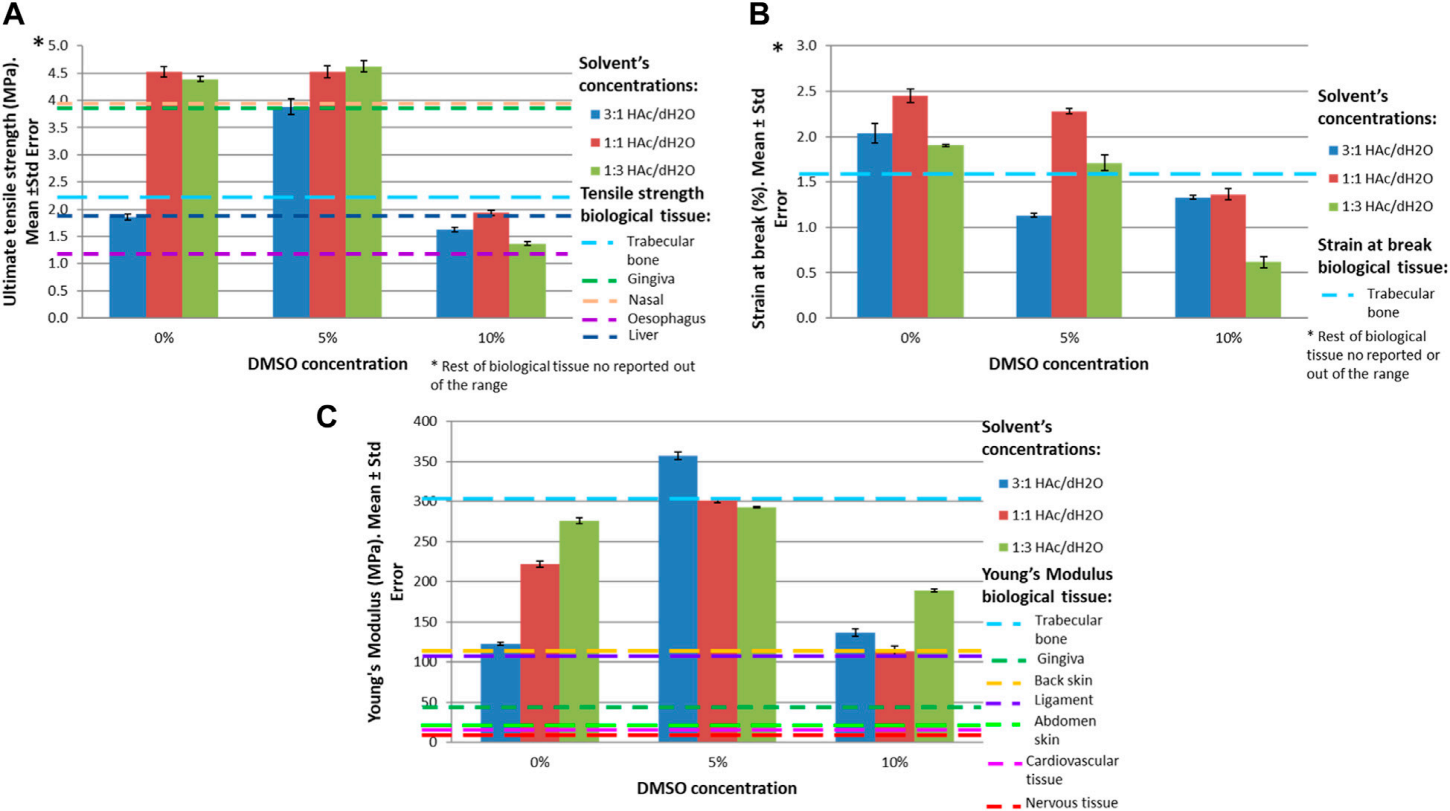
Mechanical properties for different concentrations of HAc and DMSO in comparison to biological human tissue. **(A)** Ultimate tensile strength (MPa), **(B)** Strain at break (%), **(C)** Young’s Modulus (MPa).

Figure 6 presents the mechanical properties of the different manufacture scaffolds compared to biological human tissue.

This study demonstrated that the large number of significant factors and interactions are a source of high controlled variability, therefore, by correctly managing the levels of factors and interactions, suitable results can be achieved to create scaffolds that bio-mimic an important variety of human tissues. The findings within this study quantify statistical relationships using linear regression models (Eqs 1–5) and study the importance of each factor and their interaction through a MANOVA. These studies are an excellent starting point for future research to use machine learning approaches to classify, predict and optimize manufacturing models for electrospun gelatin (Roldán et al., 2023).

### 3.2 How crosslinking affects the degradability of the scaffold, its morphology and topography

Gluteraldehyde was the selected crosslinking agent due to its low cost, strength, water resistance (Farris et al., 2010) and better cell viability reported in comparison to physical crosslinking such as dehydrothermal treatment (Gomes et al., 2013).

Non-crosslinked samples exhibited total dissolution within 1 h of incubation in PBS making the use of any biological assay and therefore the manufacturing of any implant prototype invalid. Samples crosslinked by immersion were found inviable, since they partially dissolved within 2 h of immersion in GTA. Samples crosslinked by vapor deposition with 2.5% GTA were dissolved after 3 days of incubation in PBS. 5% GTA crosslinked samples (vapor deposition) lasted 7 days before being completely dissolved. Samples exposed to 25% GTA in vapor deposition remained undegraded until day 28. Although their morphology and topography were slightly modified after performing this study, previous studies with electrospun scaffolds reported excellent cell adhesion and proliferation with values of fiber diameter between 379 ± 37 nm and 524 ± 31 nm (Chahal et al., 2016), values in the same range as the scaffolds manufactured in the current study.

Due to only the samples exposed to 25% GTA being capable of use in tissue engineering applications, only those samples are studied. However, gelatin-based crosslinked electrospun scaffolds with fast degradation rates (such as with 2.5% or 5% GTA) could be suitable for wound healing applications or drug delivery.

**FIGURE 7:**
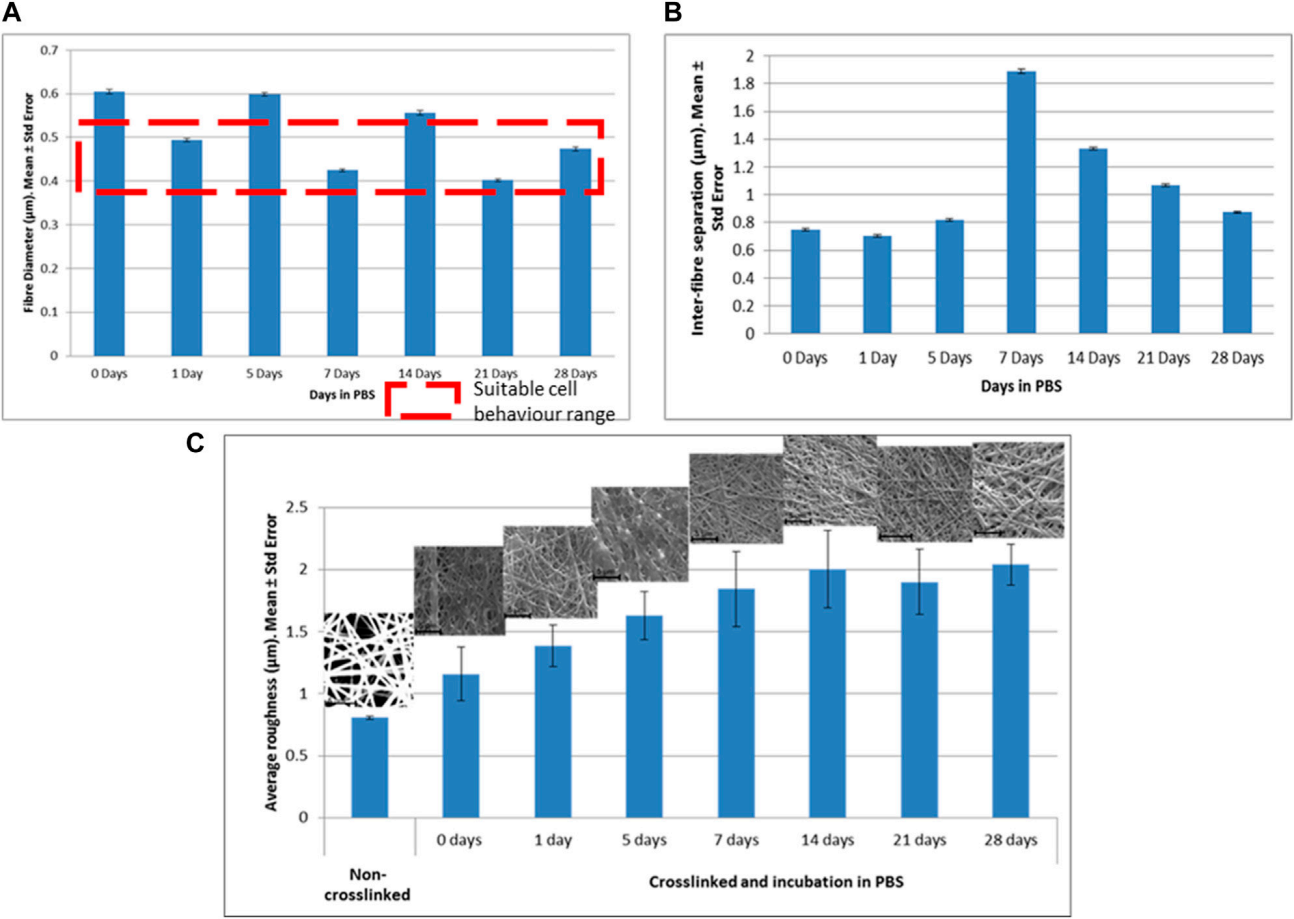
Morphological and topographical properties of crosslinked scaffolds with 25% GTA. **(A)** Fiber diameter (μm), **(B)** Inter-fiber separation (μm), **(C)** Average roughness (μm).

After a statistical analysis, it was found that there was a moderate negative correlation (−0.512) between the diameter of the fibers and days in PBS; therefore, when the incubation days increased, the diameter of the fiber decreased. A very weak positive correlation was found between the days in PBS and the inter-fiber separation (0.108). Regression models were performed for both parameters but neither the model nor the coefficients were significant. It is worth noticing that in dry conditions, the greater the fiber diameter, the greater the inter-fiber separation is, as it was demonstrated in 3.1. However, ones the scaffolds are created and immersed in PBS, the scaffolds are swollen, and the inter-fiber separation is reduced (see Figures 7A, B, day 5 vs. day 7).

The values for diameters of the fibers and inter-fiber separations for the samples exposed to 25% GTA versus the days in PBS are presented in Figure 7A, B.

The average roughness and days in PBS showed a significant positive correlation (0.836). Therefore, the roughness of the scaffold tended to increase with the presence of PBS. The regression model between those parameters and its coefficients proved to be significant (*p* < 0.05) and it is shown below 5):

$$Average\;roughness\;\left(µm\right)=1.423-0.026\;x\;days\;in\;PBS$$ (5)

This phenomenon could be due to the exposure of the fibers to salts dissolved in the PBS modifying their original topography. Figure 7C shows the values of average roughness versus days in incubation in PBS and SEM images of each scaffold.

## 4 Conclusion

Currently, combinations of gelatin with different polymers are used to create tissue engineering implants due to gelatin being an inexpensive, biocompatible and biodegradable material. However, the suitability of gelatin as a sole material to manufacturing electropun gelatin scaffolds had not previously been assessed. This study evaluated for the first time the suitability of gelatin (as a sole material) electrospun scaffolds for their use as tissue engineered implants. In order to evaluate this suitability, the influence of a crosslinking agent (GTA) and solvents such as acetic acid (HAc), distillate water (dH_2_O) and dimethyl sulfoxide (DMSO) in the morphology, topography and mechanical properties of electropun gelatin scaffolds were investigated.

This study concluded that the diameter of the fibers, inter-fiber separation, roughness and ultimate tensile strength were significantly affected by the concentration of HAc and DMSO (*p* < 0.001). It was observed that the diameter of the fibers and inter-fibers separation increased when the solvent’s concentration increased, and the roughness decreased when the concentration of DMSO increased. Mechanical properties were highly affected by the concentration of DMSO. The degradation study concluded that samples exposed to 25% GTA remained undegraded until day 28 and the achieved morphology is ideal for cell investigation.

Our gelatin-based electrospun scaffolds presented comparable mechanical properties to human tissue such as trabecular bone, gingiva, nasal periosteum, esophagus, and liver tissue, proving that electrospun scaffolds with gelatin as a sole material can bio-mimic a significant number of human tissues. This study demonstrated the importance of correctly managing the levels of factors and their interactions for developing scaffolds that bio-mimic human tissues. Further studies could be focused on developing a specific tissue by optimizing the level of factors and interactions, with the help of traditional and machine learning techniques.

## Data Availability

The original contributions presented in the study are included in the article/supplementary material, further inquiries can be directed to the corresponding author.
